# Assessment of alcohol and tobacco use in psychotherapy: validation of the german self-report ASSIST alcohol and tobacco subscales in an outpatient setting

**DOI:** 10.1016/j.abrep.2025.100634

**Published:** 2025-10-20

**Authors:** Esra Sünkel, Alla Machulska, Marie Neubert, Tobias Stalder, Tim Klucken

**Affiliations:** University of Siegen, Germany

**Keywords:** Substance use screening, Psychometric validation, Psychotherapy research, Alcohol and tobacco use

## Abstract

Alcohol and tobacco use are leading causes of preventable mortality and transdiagnostic risk factors for poor health, especially in comorbid mental disorders. Identification and treatment in German healthcare remain inadequate. The WHO ASSIST is a brief screening tool for substance use, but the German self-report version has not been systematically validated for alcohol and tobacco. Methods: In a psychotherapeutic outpatient sample (*N* = 553; mean age 34.9, *SD* = 13.4, range 18–74; 60 % female), we evaluated (i) concurrent, (ii) construct, (iii) discriminative validity, and (iv) diagnostic accuracy of the alcohol (ASSIST_A_) and tobacco (ASSIST_T_) subscales. Results: Regarding concurrent validity, ASSIST_A_ strongly correlated with AUDIT and showed weak correlation with clinical judgement, whereas ASSIST_T_ correlated with FTND but not with clinical judgement. Construct validity was demonstrated by acceptable internal consistency and small correlations with ICD-10 alcohol and tobacco diagnoses. Discriminative validity was good for both subscales. Diagnostic accuracy analyses indicated that optimal cutoff values were substantially lower than the manual thresholds (alcohol 3.5/8.5 vs. 11/27, tobacco 5.5 vs. 27). Discussion: Our results support the validity of the German ASSIST_A/T_ subscales. Comparison with established measures highlights clinical utility. ASSIST_T_‘s lack of correlation with clinical judgement suggests smoking might be under-recognized without standardized screening. We propose empirically derived cutoff values to improve sensitivity for detecting clinically relevant substance use in outpatient psychotherapy settings.

## Highlights

• First large sample validation of German ASSIST alcohol and tobacco subscales.
• Alcohol and tobacco subscales demonstrate strong validity reliability.
• Considerably lower cutoffs for alcohol (8.5) and tobacco (5.5) dependence.
• Only small-to-medium-sized associations with clinical judgement.
• German ASSIST as useful tool for standardized substance screening in psychotherapy.

## 1 Introduction

Alcohol and tobacco are among the most significant preventable health risks worldwide and impose substantial socioeconomic burdens on healthcare system (Effertz & Mann, 2013). In Europe (Peacock et al., 2018) and Germany (Rauschert et al., 2022), they remain the most widely used substances and rank among the leading contributors to disease burden, disability, and premature mortality (Global Burden of Disease 2017 Risk Factor Collaborators, 2018). Particularly concerning are the high use rates among individuals with mental disorders. More than half of individuals with mental disorders smoke tobacco (Mackay & Eriksen, 2002), and their risk of developing an alcohol use disorder is approximately twice as high as in the general population (Puddephatt et al., 2022). Strong links between mental disorders and both alcohol and tobacco use have been consistently documented (Grant et al., 2009, Puddephatt et al., 2022, Swendsen et al., 2010).

Beyond prevalence, alcohol and tobacco use in individuals with mental disorders has profound clinical implications, as the relationship between substance use and mental health is bidirectional: While alcohol and tobacco can contribute to the onset and exacerbation of mental health problems, individuals with mental disorders may also use these substances to cope with psychological distress, as outlined in the self-medication hypothesis (Khantzian, 1997). This cycle amplifies risks, as substance use and symptoms reinforce each other. Besides that, alcohol and tobacco use can also interfere with key psychotherapeutic processes. Empirical evidence links both substances to impairments in emotion regulation (Kober and Bolling, 2014, Lesage et al., 2017, McLaughlin et al., 2015, Watkins et al., 2015) and cognitive function (Curtin and Fairchild, 2003, Jellinek and McFarland, 1940, Machulska et al., 2024, Stacy and Wiers, 2010). Furthermore, they are associated with premature treatment dropout (Schawohl & Odenwald, 2018) and treatment retention (Bouchard et al., 2022, Buckman et al., 2018). In contrast, tobacco cessation has been shown to improve mental health outcomes (Taylor et al., 2014). The impact of alcohol and tobacco use on the psychotherapeutic process highlights the importance of screening for substance use at the initial psychotherapeutic contact when individuals with mental disorders first seek treatment.

Despite the well-documented risks of alcohol and tobacco use among individuals with mental disorders, systematic identification of alcohol and tobacco use remains under-implemented in psychotherapy settings (Frischknecht et al., 2021). Although national guidelines in Germany recommend standardized screening (Batra et al., 2021, Kiefer et al., 2021), the implementation of guideline-compliant diagnostics and interventions for problematic alcohol and tobacco use remains rare (Frischknecht et al., 2021). As a result, alcohol and tobacco use often remains undiagnosed in outpatient psychotherapeutic settings or is assessed solely based on clinical judgement (Frischknecht et al., 2021, Kastaun et al., 2022, Trautmann et al., 2016), despite its relevance for treatment outcomes. Several factors likely contribute to the low identification rates of cases with potentially problematic substance use patterns due to the low use of standardized screening instruments. Although mental health practitioners generally have a positive attitude toward standardized screening, practitioners often solely rely on clinical judgement and evidence-based tools are still underutilized (Revill et al., 2022). This may partly be due to practical barriers: Comprehensive screening instruments can be time-consuming, posing a challenge for psychotherapists facing time constraints (Harris et al., 2016), whereas shorter tools often focus on dependence, failing to detect risky or harmful use (McPherson and Hersch, 2000, Revill et al., 2022). Additionally, patient denial or feelings of shame in interview settings can further limit screening effectiveness (Hser et al., 1999, Stein et al., 2008). These barriers highlight the need for a brief, valid, and ecologically viable screening tool that facilitates the systematic identification of alcohol and tobacco use without placing additional burden on psychotherapists.

The Alcohol, Smoking, and Substance Involvement Screening Test (ASSIST; World Health Organization ASSIST Working Group, 2002) directly addresses these challenges: Developed by the World Health Organization (WHO), it offers an efficient, standardized approach to substance use screening that reduces reliance on unstructured clinical judgment and avoids the time demands of using multiple substance-specific instruments. By assessing a range of substances, including alcohol and tobacco, within a single, brief diagnostic tool, the ASSIST provides a practical and time-saving solution tailored to the needs of routine psychotherapeutic settings. More recently, a shorter version (ASSIST-11) has been developed and cross-culturally validated, yielding a unidimensional score across substances for general screening purposes (Lee et al., 2023, Horváth et al., 2023), while the original ASSIST retains substance-specific scores that support more differentiated assessments. Validation studies of the original English-language ASSIST demonstrate strong psychometric properties for the alcohol and tobacco subscales, with high correlations with the Alcohol Use Disorders Identification Test (AUDIT; *r* = 0.82; Humeniuk et al., 2008) and the Fagerström Test for Nicotine Dependence (FTND; *r* = 0.85; Newcombe et al., 2005), respectively. The alcohol subscale further shows good discriminative validity (sensitivity range: 80 % − 83 %, specificity range 71 % − 79 %; Humeniuk, 2008). In contrast, the tobacco subscale has not yet been examined regarding discriminative validity (e.g., Humeniuk et al., 2008). Test-retest reliability was high for both subscales (*r* = 0.80 − 0.83; Henrique et al., 2004, Tiburcio Sainz et al., 2016).

Despite extensive validation of the English version (e.g. Humeniuk et al., 2006, Newcombe et al., 2005), research on its German self-report adaptation remains limited. To date, only one preliminary pilot study has examined the German version of the ASSIST. While Schütz et al. (2005) developed a German translation of the ASSIST in accordance with WHO guidelines, their study focused primarily on linguistic adaptation and feasibility in general medical treatment facilities. While the study confirmed basic comprehensibility and usability, substantial gaps regarding questionnaire validation remained: Schütz et al. (2005) (i) did not assess any psychometric properties, such as reliability or validity, (ii) lacked any detailed analysis of individual substance subscales, particularly for alcohol and tobacco, (iii) did not include comparisons with established reference measures or routine clinical assessment methods, such as psychotherapists’ evaluations of current substance use, and (iv) only grounded their findings on a small sample size (*N* = 17), which might thus limit the generalizability of findings. The present study seeks to address the outlined limitations in a considerably larger sample size. Specifically, we evaluate the psychometric properties of the alcohol and tobacco subscales and examine their agreement with clinical judgment, aiming to evaluate whether the ASSIST may serve as a suitable and practical screening tool in outpatient psychotherapy settings for individuals with mental disorders.

In detail, the ASSIST alcohol (ASSIST_A_) and tobacco (ASSIST_T_) subscales were tested against established and clinical guideline conform instruments, practice-oriented clinical judgement by licensed psychotherapists, as well as gold-standard International Statistical Classification of Diseases and Related Health Problems, 10^th^ revision (ICD-10; World Health Organization, 2004) dependence diagnoses assessed via structured clinical interviews. Precisely, the study pursued the following objectives: First, to assess concurrent validity, referring to the extent to which a test correlates with other established measures that assess the same construct, by comparing the ASSIST_A_ and ASSIST_T_ subscales with established measures as the Alcohol Use Disorders Identification Test (AUDIT; World Health Organization et al., 2001) and the Fagerström Test for Nicotine Dependence (FTND; Fagerström, 1978), as well as therapists’ clinical judgement (CJ_A,T_). Second, to evaluate construct validity through internal consistency and correlations between ASSIST-based dependence constructs (Schütz et al., 2005) and ICD-10-based substance dependence diagnoses. Third, to examine discriminative validity, that is, the ability to differentiate between individuals with and without clinically relevant alcohol or tobacco use, based on external clinical criteria. Fourth, to determine diagnostic accuracy, including sensitivity, specificity, and optimal cutoff scores for detecting problematic use in outpatient psychotherapy settings. This methodological approach may facilitate an ecologically valid evaluation of the ASSIST_A_ and ASSIST_T_ subscales’ applicability in everyday clinical contexts but also in research.

## 2 Methods

### 2.1 Participants

The study was preregistered on OSF (Sünkel et al., 2024). The total sample consisted of 553 individuals with mental disorders (affective disorders: 44 %; anxiety disorders: 29 %; adjustment disorder: 27 %; substance use disorders: 9 %; somatoform disorders: 7 %; attention deficit hyperactivity syndrome: 5 %: post traumatic stress disorder: 5 %; others: 10 %, including obsessive–compulsive disorder, eating disorders, personality disorders). Importantly, participants were not specifically recruited for substance use problems, reflecting the typical heterogeneity of patients in routine psychotherapeutic care. The patients visited the University of Siegen’s outpatient psychotherapeutic clinic for an initial psychotherapeutic consultation. The participants’ mean age was 34.86 years (*SD* = 13.37; ranging 18–74 years). Of these, 332 (60 %) identified as female and 221 (40 %) as male. All participants presented for an initial consultation and provided written informed consent. Inclusion criteria required sufficient German language proficiency and 18 years of age. These broad inclusion criteria are intended to enhance external validity by capturing a wide and realistic range of outpatients. Standardized self-report data (ASSIST, AUDIT and FTND) were available for the full sample. Due to practical constraints in the clinical setting, additional diagnostic data were collected from partially overlapping subsamples. About one third of participants (subsample A; *n* = 221) were rated by licensed psychotherapists (clinical judgement; CJ_A,T_), while another third (subsample B; ICD_A_: *n* = 209, ICD_T_: *n* = 207) underwent structured ICD-10 interviews conducted by independent trained diagnosticians, who were different from the treating therapists. For approximately one third of the sample (subsample C), both clinical judgement and ICD-10 diagnoses were available.

### 2.2 Study design & procedure

The research project was approved by the ethics committee of the University of Siegen (reference number: ER_24_2022). After providing informed consent and completing administrative forms, patients attended an initial psychotherapy session, during which licensed therapists conducted a brief anamnesis and provided a clinical judgement rating of patients’ alcohol (CJ_A_) and tobacco (CJ_T_) risk. These ratings (subsamples A and C) were recorded before the second session to avoid recall bias. At the second session, all patients completed self-report questionnaires (ASSIST, AUDIT, FTND). Subsequently, trained diagnosticians – independent from therapists of the first session – conducted semi-structured ICD-10 interviews assessing alcohol (ICD_A_) and tobacco (ICD_T_) dependence (subsamples B and C). Data assessment took place between October 2023 and March 2025.

### 2.3 Measures

German version of the Alcohol, Smoking and Substance Involvement Screening Test (ASSIST; Schütz et al., 2005): The ASSIST, developed by the WHO, is a brief screening tool for identifying and assessing substance use, including tobacco and alcohol. The ASSIST enables a dimensional assessment of substance use beyond categorical diagnoses. It is applicable as interview or self-report (World Health Organization, 2002). The ASSIST takes approximately 5–10 min to complete and was used in this study as a self-report questionnaire. It has demonstrated strong psychometric properties in both interview and self-report versions (e.g., Humeniuk et al., 2008, Newcombe et al., 2005, Tiburcio Sainz et al., 2016), including good test–retest reliability (McNeely et al., 2014) and high patient compliance (Spear et al., 2016). For each substance ever used (lifetime use), seven follow-up items are provided; if lifetime use is denied, no further questions follow. The follow-up items assess aspects such as frequency of use, cravings, problems related to use, and concern from others. Responses are given on a 5-point Likert scale ranging from 0 (*never*) to 4 (*daily or almost daily*). The ASSIST allows for the assessment of various substance use–related facets:

*Substance involvement scores* are calculated by summing the responses to the seven substance-specific items, excluding the initial lifetime use question and the optional item on injection drug use and range from 0 − 31 for tobacco and 0–39 for other substances. Risk categories are defined as low (0–10 for alcohol; 0–3 for tobacco), moderate (11–26; 4–26), and high (≥ 27 for both; Schütz et al., 2005).

In addition, a *dependence index* can be calculated by summing five items reflecting core features of dependence in line with the ICD-10-dependence criteria: frequent use, craving, continued use despite harm, unsuccessful control efforts, and perceived need to function (Humeniuk et al., 2006). While not diagnostic, this index corresponds to ICD-10 criteria and enables dimensional assessment of likely dependence severity.

Alcohol Use Disorder Identification Test (AUDIT; World Health Organization et al., 2001): The AUDIT, a brief 10-item questionnaire assessing alcohol consumption and related problems, is considered suitable for all clinical settings to assess alcohol use patterns (Haber et al., 2009, Reinert and Allen, 2007, Zhang et al., 2017) and has demonstrated solid psychometric properties across genders and cultural subgroups (Horváth et al., 2023, Lee et al., 2023). The AUDIT assesses alcohol consumption, dependence symptoms, and related harms over the past 12 months. For this study, the German self-report version (Dybek et al., 2006), with an editing time of approximately 5 min was used. Like the original English version, it is a 10-item questionnaire and all items are rated on 5-point Likert-type scales. The AUDIT conducts a *total score*, ranging from 0 to 40, and is calculated by summing all item scores. A score of 0 indicates abstinence. According to the WHO (2001), scores of 1–7 reflect low-risk consumption, 8–14 indicate harmful consumption, and ≥ 15 suggest a high likelihood of alcohol dependence. These cutoffs were used to test discriminative validity of the ASSIST alcohol subscale.

Fagerström Test for Nicotine Dependence (FTND; Fagerström, 1978; German Version: Bleich et al., 2002): The FTND is a widely used six-item assessment that measures the severity of physical nicotine dependence. The items address cigarette consumption, compulsion to use, and dependence based on current smoking behavior and dependence symptoms, without a fixed retrospective time frame. It is brief questionnaire, with four items scored on a binary scale (0 = “no”, 1 = “yes”) and the remaining two items scored on an ordinal scale from 0 to 3.

The FTND conducts a *total score* that ranges from 0 to 10, with higher scores indicating greater nicotine dependence. Scores of 0–2 indicate low dependence, 3–5 moderate dependence, 6–7 high dependence, and 8–10 very high dependence (Fagerström, 1978). These cutoffs were used to test discriminative validity of the ASSIST tobacco subscale.

Clinical Judgement: This variable employed two items that aimed to evaluate the risk for alcohol and tobacco use disorder, respectively, as anticipated by the therapist (e. g., *“How do you anticipate this patient’s risk of an alcohol-related [tobacco-related] disorder: Low risk, moderate risk, or high risk?”*). Responses were categorized according to the ASSIST’s risk levels: low, moderate, and high. Two ordinal variables of clinical judgement were determined, one for risk for alcohol-related disorder (CI_A_) and one for risk for tobacco-related disorder (CI_T_), with scores of 0 (indicating low risk), 1 (indicating moderate risk), and 2 (indicating high risk).

Semi-structured clinical interview for ICD − 10 criteria for dependence: A semi-structured interview assessed the six ICD-10 dependence criteria (World Health Organisation, 2004). The criteria were queried in a binary “yes”/“no” format for both alcohol and tobacco (e.g., “*During the last 12 months, has there been a strong desire or compulsion to consume alcohol?*”) and rated by the trained diagnostician based on the patient’s answer. If more than three items were answered with “yes,” the criteria for dependence were considered met. Based on the ICD-10 interview, two binary variables were created: one for alcohol (ICD_A_) and one for tobacco (ICD_T_) dependence, indicating whether dependence criteria were met or not.

### 2.4 Data analysis

All statistical analyses were conducted using IBM SPSS, version 27.0. An alpha level of 0.05 was used for all statistical tests. Table 1 summarizes the assessed validity types and metrics.

**Table 1:** Overview of validation types, methods and reported metrics.

Validation Type & Criteria	Method	Reported metric
***(i) Concurrent validity***		
ASSIST_A_ ↔ AUDIT, CJ_A_	Pearson correlation analysis	*r, p-*value
ASSIST_T_ ↔ FTND, CJ_T_		

***(jj) Construct validity***		
Internal consistency of ASSIST_A_ and ASSIST_T_	Cronbach’s alpha	Cronbach’s *α*
ASSIST alcohol dependence construct ↔ ICD_A_	Point-biserial correlations	*r, p-*value
ASSIST tobacco dependence construct ↔ ICD_T_		

***(iii) Discriminative validity***		
ASSIST alcohol risk groups (AUDIT reference)	ANOVA (Games-Howell)	*F*(*df*), *p-*value, *η*2
ASSIST tobacco risk groups (FTND reference)	ANOVA (Tukey HSD)	

***(iv) Diagnostic accuracy***		
ASSIST_A_ − risky alcohol use (reference: AUDIT ≥ 8)	ROC curve analyses	*AUC* value*,* 95 % *CI,* Sensitivity, Specificity, Youden-Index *J,* cutoff-scores
ASSIST_A_ − alcohol dependence (reference: ICD-10)		
ASSIST_T_ − moderate dependence (reference: FTND ≥ 3)		
ASSIST_T_ − tobacco dependence (reference: ICD-10)		

*Note*. ASSIST_A,T_ = Alcohol, Smoking and Substance Involving Test involvement.

score for alcohol **(A)** and tobacco **(T)**; AUDIT = Alcohol Use Disorder Identification Test; FTND = Fagerström Test for Nicotine Dependence; CJ_A_,_T_ = Clinical judgement for alcohol **(A)** and tobacco **(T)** related disorders; ICD_A,T_ = ICD-10 alcohol **(A)**/tobacco **(T)** dependence diagnosis; ROC = Receiver Operating Characteristics; *AUC* = Area under the curve; *df* = degrees of freedom.

First, concurrent validity, referring to the extent to which a test correlates with other established measures that assess the same construct, was examined by calculating two-tailed Pearson correlations between the ASSIST subscale scores for alcohol and tobacco and scores of the AUDIT, FTND, and therapists’ clinical judgement (CJ_A_ and CJ_T_). Large correlations between ASSIST scores and external measures were interpreted as indicators of high concurrent validity, using Cohen’s (1988) benchmarks: small (*r* = 0.10), medium (*r* = 0.30), and large (*r* = 0.50) correlations.

Second, construct validity was assessed in two steps: (1) by evaluating internal consistency using Cronbach’s alpha, and (2) by examining correlations between ASSIST-based dependence constructs and ICD-10-based substance dependence diagnoses using Pearson correlations.

Third, discriminative validity, referring to the ability of the instrument to differentiate between clinically relevant subgroups, was tested separately for alcohol and tobacco. For alcohol, participants were divided into three groups (low-, moderate-, and high-risk use) based on AUDIT cutoff scores, as suggested in the AUDIT manual (WHO, 2001). Group differences in ASSIST alcohol mean scores were analyzed using Welch’s ANOVA and post-hoc Games-Howell tests due to violations of normality (Shapiro-Wilk tests: *ps* < 0.002) and variance homogeneity (Levene’s test: *F*(2,540) = 41.3, *p* < 0.001). For tobacco, participants were grouped according to their FTND scores into four levels of nicotine dependence. One-way ANOVA with post-hoc Tukey tests was applied to assess mean differences in ASSIST tobacco scores, with normality assumptions met for high and very high dependence levels (Shapiro-Wilk *ps* = 0.954/.332), but not for low and moderate levels (*ps* = 0.005/<.001); variance homogeneity was confirmed (Levene’s test: *F*(3,527) = 0.79, *p* = 0.501). Significant group differences were interpreted as evidence of good discriminative validity.

Fourth, diagnostic accuracy was assessed to determine the ability of the ASSIST alcohol (ASSIST_A_) and tobacco (ASSIST_T_) subscales to identify problematic use in an outpatient psychotherapy setting. Receiver Operating Characteristic (ROC) curve analyses were conducted for both subscales, focusing on the area under the curve (AUC), sensitivity, specificity, and optimal cutoff scores based on the Youden index (Perkins & Schisterman, 2006).

For ASSIST_A_, diagnostic accuracy was evaluated with two reference standards: (1) to differentiate low-risk from risky alcohol use, the AUDIT cutoff for moderate risk (AUDIT ≥ 8) was applied; (2) to detect alcohol dependence, the presence of ICD-10-based alcohol dependence (ICD_A_) served as the gold standard.

For ASSIST_T_, ROC analyses were used to distinguish between different levels of tobacco dependence. The presence of ICD-10-based tobacco dependence (ICD_T_) was used as the gold standard. Additionally, to differentiate between low and moderate dependence, the FTND cutoff score for moderate dependence (FTND ≥ 3) was used as a reference. Confidence intervals (95 %) were calculated for all ROC analyses with a significance threshold of *p* < 0.05. *AUC* scores were interpreted using Swets’ (1996) guidelines: *AUC* < 0.70 indicates low diagnostic accuracy, *AUC* = 0.70–0.90 reflects moderate accuracy, and *AUC* ≥ 0.90 represents high diagnostic accuracy. This methodological approach aims to provide an ecologically valid evaluation of the ASSIST’s applicability in both clinical practice and research contexts.

## 3 Results

### 3.1 Concurrent validity: correlations with AUDIT, FTND and clinical judgement

As mentioned, to examine concurrent validity, correlations between the ASSIST subscale scores and established measures were analyzed. Table 2 shows sample sizes, means, standard deviations, and the correlation matrix of the continuous measures. ASSIST_A_ and AUDIT scores were strongly positively correlated (*r* = 0.82, *p* < 0.001), suggesting good convergent validity for the assessment of alcohol use. A weak, but significantly positive correlation was also found between ASSIST_A_ and CJ_A_ scores (*r* = 0.21, *p* = 0.002). For tobacco use, ASSIST_T_ also showed a significant moderate to strong correlation with FTND scores (*r* = 0.66, *p* < 0.001). In contrast, no significant correlation was found between ASSIST_T_ and CJ_T_ (*r* = 0.12, *p* = 0.084).

**Table 2:** Means, medians (in brackets), standard deviations and Pearson correlations.

	*n*	*M (Mdn)*	*SD*	1	2	3	4	5	6
1. **ASSIST_A_**	553	5.35 (3)	6.43	1.00					
2. **ASSIST_T_**	553	5.41 (0)	7.89	0.30**	1.00				
3. **AUDIT**	543	4.7 (3)	5.07	0.82**	0.30**	1.00			
4. **FTND**	531	0.65 (0)	1.67	0.12**	0.66**	0.12**	1.00		
5. **CJ_A_**	221	0.63 (0)	1.05	0.21**	0.07**	0.21**	0.11	1.00	
6. **CJ_T_**	221	0.66 (0)	0.84	−0.01	0.12	−0.05	0.14*	0.36**	1.00

*Note.* ASSIST_A_ = German version of the Alcohol, Smoking and Substance Involvement Screening Test, subscale for alcohol; ASSIST_T_ = German version of the Alcohol, Smoking and Substance Involvement Screening Test, subscale for tobacco; AUDIT = Alcohol Use Disorder Identification Test; FTND = Fagerström Test for Nicotine Dependence CJ_A_,_T_ = Clinical judgement for alcohol and tobacco related disorders; * *p* < 0.05. ** *p* < 0.01.

### 3.2 Construct validity: internal consistency and associations with ICD-10 diagnoses

To examine construct validity, internal consistency of the ASSIST subscales was assessed using Cronbach’s alpha, and associations with ICD-10-based substance dependence diagnoses (ICD_A_, ICD_T_) were analyzed. The ASSIST_A_ demonstrated acceptable internal consistency (Cronbach’s *α* = 0.77) and showed a small correlation with the presence of alcohol dependence according to ICD-10 (ICD_A_; *r* = 0.25, *p* < 0.001, *n* = 209). The ASSIST_T_ demonstrated good internal consistency (Cronbach’s *α* = 0.84). The ASSIST_T_ also showed a small correlation with the presence of tobacco dependence according to ICD-10 (ICD_T_; *r* = 0.18*, p* = 0.009, *n* = 207). These small correlations indicate that the ASSIST subscales are statistically associated with ICD-10-based diagnoses, even if correlations values are generally low.

### 3.3 Discriminative validity: group differences based on AUDIT and FTND risk levels

**Fig. 1:**
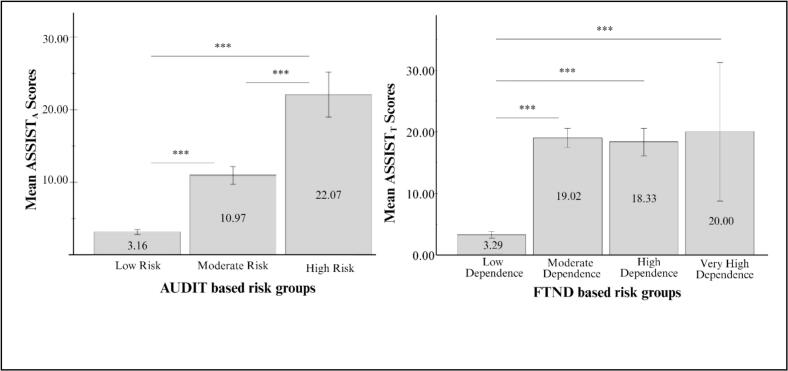
Mean ASSIST_A_ scores across AUDIT-based alcohol risk groups (low, moderate, high) and mean ASSIST_T_ scores across FTND-based tobacco dependence groups (low, moderate, high, very high). Note. Error bars represent 95 % confidence intervals. *** p < 0.001.

To evaluate discriminative validity, ASSIST scores were compared across groups defined by risk levels on established measures. As shown in Fig. 1, the ASSIST_A_ significantly differentiated between individuals with varying levels of alcohol-related risk based on the AUDIT. Welch’s ANOVA revealed significant group differences between low-risk, moderate-risk, and high-risk individuals (*F(*2, 56.64) = 145.4, *p* < 0.001). Specifically, Games-Howell post hoc tests revealed that patients classified as low-risk (*M* = 3.16, *SD* = 3.60) scored significantly lower than those in the moderate-risk group (M = 10.96, SD = 5.91), with a mean difference of 7.80 (95 % *CI* [6.27, 9.33], *p* < 0.001). The low-risk group also scored significantly lower than the high-risk group (*M* = 22.07, *SD* = 7.80), with a mean difference of 18.92 (95 % *CI* [15.17, 22.66], *p* < 0.001). Additionally, the moderate-risk group scored lower than the high-risk group, with a mean difference of 11.12 (95 % *CI* [7.15, 15.09], *p* < 0.001).

In contrast, ASSIST_T_ primarily distinguished between patients with low and more severe nicotine dependence as defined by the FTND (*F*(3, 527) = 132.09, *p* < 0.001; see Fig. 1). Tukey HSD post hoc tests showed that the FTND-low group (*M* = 3.29, *S*D = 6.09) scored significantly lower than the moderate (*M* = 19.02, *SD* = 5.18; mean difference = 15.74, 95 % *CI* [13.56, 17.91], *p* < 0.001), high (*M* = 18.33, *SD* = 4.03; mean difference = 15.04, 95 % *CI* [11.47, 18.60], *p* < 0.001), and very high group (*M* = 20.00, *SD* = 7.07; mean difference = 16.71, 95 % *CI* [9.66, 23.76], *p* < 0.001). No significant differences were found among the three higher dependence groups (*p* = 0.980, *p* = 0.989, and *p* = 0.960, respectively).

### 3.4 Diagnostic accuracy: sensitivity, specificity, and cutoff scores based on AUDIT, FTND and ICD-10

To assess diagnostic accuracy, sensitivity, and specificity, Youden indices were calculated for various ASSIST_A_ and ASSIST_T_ cutoff scores, respectively. Table 4 demonstrates sensitivity, specificity, and Youden-indices for different ASSIST_A_ cutoff values for the identification of risky alcohol users according to the AUDIT and dependent users as indicated by ICD-10 dependence diagnosis (ICD_A_).

ASSIST_A_ revealed excellent diagnostic accuracy for identifying risky alcohol use: ROC curve analysis for identification of risky alcohol use (in comparison to low-risk use) based on the AUDIT cutoff score ≥ 8 revealed a high area under the curve value of *AUC* = 0.92 (*SD* = 0.01; 95 % - *CI *[.89, 0.94]). The ASSIST_A_ *AUC* = 0.92 reached statistical significance (*p* < 0.001), implying that the groups, low risk versus at-risk, were statistically disparate. The cutoff value of 3.5 yielded the highest Youden index ($J_{\max}=\;.68),$ with a very high sensitivity of 97 % and good specificity of 72 %.Compared to the high accuracy for identifying risky alcohol use, diagnostic performance for detecting alcohol dependence (versus non-dependence, indicated ICD_A_) was lower but remained within a moderate and acceptable range.

ROC curve analysis for identification of dependent alcohol users revealed an area under the curve value of *AUC* = 0.79 (*SD* = 0.10; 95 % − *CI *[.59, 0.98]) and reached statistical significance (*p* = 0.004). In regard to dependence, the ASSIST_A_ cutoff value of 8.5 offered the highest Youden-index (Youden’s $J_{\max}=\;.55).\;$ The ASSIST_A_ cutoff of 8.5 presented with a sensitivity rate of 75 % and a specificity rate of 80 % (see Table 3).

**Table 3:** Sensitivity and specificity for different ASSIST_A_ cutoff values for risky alcohol use and dependence.

Cutoff value	ASSIST_A_						
	Risky use (AUDIT; n = 543)				Dependence (ICD-10; n = 209)		
	Sensitivity (%)	Specificity (%)	J		Sensitivity (%)	Specificity (%)	J
**1.0**	0.99	0.31	0.30		0.88	0.25	0.12
**2.5**	0.99	0.54	0.53		0.88	0.45	0.32
**3.5**	**0.97**	**0.72**	**0.68**		0.88	0.57	0.45
**5.5**	0.86	0.79	0.66		0.75	0.65	0.47
**7.5**	0.71	0.90	0.61		0.75	0.78	0.53
**8.5**	0.69	0.92	0.61		**0.75**	**0.80**	**0.55**
**9.5**	0.64	0.95	0.59		0.63	0.81	0.43
**12.5**	0.49	0.97	0.47		0.63	0.86	0.49
**14.5**	0.36	0.98	0.35		0.50	0.90	0.41
**17.5**	0.27	0.99	0.26		0.38	0.93	0.31
**21.5**	0.16	1.00	0.16		0.25	0.96	0.21
**23.5**	0.13	1.00	0.12		0.25	0.98	0.23
**26**	−^a^	−	−		0.13	0.99	0.12
**27.5**	0.05	0.10	0.05		−^a^	−	−
**31.0**	0.04	1.00	0.04		0.00	1.00	0.00

*Note.*^a^ Sensitivity and specificity values are calculated based on observed data for each cutoff, leading to missing values for certain cutoff scores. *J* = Youden-index.

Table 4 demonstrates sensitivity, specificity, and Youden-indices for different ASSIST_T_ cutoff values for the identification of low versus moderate dependent tobacco users according to the FTND and dependent (versus non-dependent) users as indicated by ICD-10 dependence diagnosis (ICD_T_). ROC analysis for identifying moderate or more severe nicotine dependence (FTND ≥ 3) showed very good diagnostic performance (*AUC* = 0.94, (*SD* = 0.01; 95 % − *CI *[.92, 0.97])). Hence, the model reached statistical significance (*p* < 0.001). As reported in Table 4, ASSIST_T_ cutoff scores between 8.5 and 10.5 were identified as the best fit for the Youden index in the identification of moderate dependent users (as assessed by FTND; *J* = 0.83). The cutoff score of 8.5 showed higher sensitivity (98 %) than the cutoff score of 10.5 (97 %).

**Table 4:** Sensitivity and specificity for different ASSIST_T_ cutoff values for forms of tobacco dependence.

Cutoff value	ASSIST_T_						
	Moderate dependence (FTND; n = 543)				Dependence (ICD-10; n = 209)		
	Sensitivity ( %)	Specificity ( %)	J		Sensitivity ( %)	Specificity ( %)	J
1.0	0.98	0.66	0.64		0.57	0.61	0.18
**2.5**	0.98	0.71	0.69		0.57	0.65	0.23
**3.5**	0.98	0.75	0.74		0.52	0.68	0.20
**4.5**	0.98	0.76	0.74		0.52	0.68	0.21
**5.5**	0.98	0.78	0.77		**0.50**	**0.72**	**0.22**
**6.5**	0.98	0.82	0.80		**0.48**	**0.75**	**0.22**
**7.5**	0.98	0.83	0.81		0.45	0.75	0.2
**8.5**	**0.98**	**0.84**	**0.83**		0.45	0.76	0.21
**9.5**	0.97	0.85	0.82		0.43	0.76	0.19
**10.5**	**0.97**	**0.86**	**0.83**		0.40	0.76	0.17
**11.5**	0.95	0.87	0.82		0.40	0.78	0.18
**14.5**	0.89	0.91	0.80		0.36	0.83	0.19
**21.5**	0.29	0.98	0.27		0.14	0.98	0.12
**23.5**	0.17	0.99	0.15		0.07	0.99	0.08
**24.5**	0.11	0.99	0.10		0.07	1.00	0.06
**27.5**	0.05	0.99	0.04		−	−	−
**28.5**	0.02	1.00	0.01		0.02	1.00	0.02

*Note.*^a^ Sensitivity and specificity values are calculated based on observed data for each cutoff, leading to missing values for certain cutoff scores. *J* = Youden-index.

However, for identifying ICD-10-based tobacco dependence (ICD_T_; in comparison to non-dependence), diagnostic accuracy was lower than for identifying low- versus moderate dependence (*AUC* = 62. (*SD* = 0.02; 95 % − *CI *[.52, 0.73])), though still reached statistical significance (*p* = 0.017). For dependence assessed by ICD_T_, ASSIST_T_ cutoff scores of 5.5 and 6.5 maximized the sum of sensitivity and specificity (*J* = 0.21). The cutoff score of 5.5 showed higher sensitivity (50 %) compared to the cutoff score of 6.5 (48 %).

## 4 Discussion

The present study sought to validate the self-report version of the WHO ASSIST alcohol and tobacco subscales in an outpatient sample receiving psychotherapy for primary mental disorders by evaluating against a variety of diagnostic standards, including established screening instruments (i.e., FTND and AUDIT), the commonly used practice-oriented standard of clinical judgment, and gold-standard ICD-10-based diagnoses.

The results support the utility of the ASSIST_A_ and ASSIST_T_ subscales as time-efficient and dimensional diagnostic tools in outpatient mental health settings. Particularly for the alcohol subscale, findings indicated strong psychometric properties across all validation indices. In the context of nicotine use, the ASSIST_T_ also showed good overall performance, however, its ability to detect ICD-10-defined tobacco dependence was notably lower and clinical judgment did not significantly correlate with ASSIST_T_ scores, as further discussed below.

## 5 Internal consistency and associations with established measures

The ASSIST_A_ and ASSIST_T_ showed good internal consistency, robust concurrent validity with established instruments (AUDIT and FTND, respectively), and meaningful associations with clinical diagnoses and clinical judgement. Diagnostic accuracy was especially high for identifying risky alcohol use and alcohol dependence. ASSIST_T_ performed well in relation to FTND-based dependence but showed limited accuracy regarding ICD-10 dependence classifications.

ASSIST_A_ demonstrated high internal consistency and excellent concurrent validity, with strong correlations with the AUDIT as well as therapists' clinical judgement. Discriminative validity was also high, as the ASSIST_A_ effectively differentiated between low, moderate, and high-risk alcohol users based on AUDIT-based risk groups. ROC analyses confirmed excellent diagnostic accuracy. Interestingly, the optimal cutoff values for risky alcohol (3.5) and alcohol dependence (8.5) identified in our sample were considerably lower than those suggested in the original ASSIST manual (11/27), indicating a need for context-specific thresholds that enhance sensitivity in outpatient mental health settings, as further discussed below. Overall, these findings add to the growing evidence base supporting dimensional alcohol screening approaches in mental health care, as reflected in diagnostic systems like the DSM-5 (American Psychiatric Association, 2013, Hasin et al., 2013).

ASSIST_T_ demonstrated strong correlations with FTND scores and high internal consistency, indicating good concurrent and construct validity. It effectively distinguished between individuals with low and more severe tobacco dependence, yet showed limited sensitivity to finer gradations among higher dependence levels. ROC analyses revealed excellent diagnostic accuracy for identifying moderate or greater tobacco dependence based on FTND criteria, though accuracy was notably lower when ICD-10 dependence was used as the reference standard. As with ASSIST_A_, optimal cutoffs for tobacco dependence were lower in this sample than those in the ASSIST manual (5.5–6.5 vs. 27). Interestingly, ASSIST_T_ scores did not significantly correlate with therapists’ clinical judgement, as further discussed below.

In summary, these findings suggest that the ASSIST alcohol and tobacco subscales adequately captures substance involvement compared to established guideline-based measures. Internal consistency, concurrent validity, and discriminative validity were supported, particularly for the alcohol subscale. Diagnostic accuracy analyses confirmed that the ASSIST can reliably identify substance-related risks, although optimal cutoff scores were lower than those proposed in the original manual, as further outlined below. While the tobacco subscale exhibited somewhat lower discriminatory power, it still provided meaningful clinical information, particularly when aligned with dimensional models such as the FTND.

The findings of this study are consistent with those of previous validation studies (e.g., Humeniuk et al., 2008), thereby further supporting the utility of the ASSIST in identifying substance-related risks in clinical practice. The present study extends the findings of previous research by demonstrating, for the first time, the validity and effectiveness of the ASSIST in an outpatient psychotherapeutic sample. This is of particular relevance to diagnostic practice in such settings, as substance use disorders are known to be highly prevalent and linked to mental disorders (Grant et al., 2009, Puddephatt et al., 2022, Swendsen et al., 2010). The ability to reliably assess substance involvement in this population therefore represents a significant step towards enhancing clinical care. Importantly, certain findings, particularly regarding discrepancies with clinical judgement and optimal cutoff scores warrant further consideration, as discussed below.

### 5.1 Associations with clinical judgement

One of the most notable findings was the low correlation between the ASSIST_T_ and therapists' clinical judgement for tobacco use which failed to reach statistical significance. This relatively low correspondence may reflect a broader clinical phenomenon: tobacco use is often perceived as a secondary issue in mental health settings or rated as rather a “lifestyle problem” than a lethal condition, and may thus be underestimated in routine assessments (Batra et al., 2021), despite its well-established negative impact on mental health outcomes (Taylor et al., 2014). In the context of psychotherapy, tobacco use can impair concentration, increase impulsivity, reduce reward sensitivity and cause emotional dysregulation (Epping-Jordan et al., 1998, Hughes et al., 2004, Lesage et al., 2017), all of which are key factors in regard to the psychotherapeutic process. Therefore, integrating tobacco use more systematically into diagnostic and treatment planning can be considered essential for improving psychotherapeutic outcomes. Structured tools like the ASSIST can complement clinical judgement by offering a standardized, dimensional approach to detecting risky tobacco use, especially in time-constrained outpatient settings where such issues might otherwise be missed (Harris et al., 2016).

To a lesser extent, this also holds for alcohol use, as evidenced by the also weak correlation between clinical judgement and ASSIST_A_. Although alcohol consumption is generally recognized as a clinical concern in psychotherapy, the correlation between clinical judgment and ASSIST_A_ scores was also weak, indicating potential gaps in the identification of problematic use. Wilson et al. (2004) found that clinicians often underdetect milder or subthreshold alcohol use disorders, particularly when patients do not exhibit clear functional impairments or overt symptoms, highlighting the challenges of relying solely on clinical judgement. At the same time, the strong correlation between the ASSIST_A_ and the AUDIT, an established screening instrument, supports the validity of the ASSIST_A_. Therefore, the low correlation of ASSIST_A_ and clinical judgement might be more likely to reflect a limitation of clinical judgement rather than a shortcoming of the ASSIST_A_ itself.

### 5.2 Cutoff points for screening in outpatient settings

While the exploration of cutoff values was not a predefined objective of this study, the observed discrepancies between manual-recommended thresholds and empirically optimal scores emerged as an important supplemental finding. The ASSIST manual thresholds are intended as guidance for categorizing risk levels (e.g., low, moderate, high; WHO Assist Working Group (2002)), rather than as empirically validated diagnostic criteria. However, our results suggest that, within outpatient psychotherapeutic settings, substantially lower cutoff scores may be more appropriate for identifying individuals at risk of problematic alcohol and tobacco use.

For alcohol use, an empirically derived cutoff of 3.5 yielded greater sensitivity for detecting risky consumption than the manual’s recommended threshold of 11, which is used to distinguish between low and moderate risk. This is particularly relevant in outpatient psychotherapy, where even low levels of alcohol use may compromise treatment efficacy (e.g., through impaired cognitive control; Curtin & Fairchild, 2003) and where subthreshold use can still lead to clinically significant consequences (Goldstein et al., 2009). Similarly, for alcohol dependence, the best-performing cutoff in our study was 8.5, which is considerably lower than the ASSIST manual’s high-risk threshold of 27. Although the manual’s threshold is not intended as a strict diagnostic marker but rather as a signal for further assessment, our finding aligns with prior studies reporting lower thresholds for detecting alcohol dependence (Adlard et al., 2023, Tiburcio Sainz et al., 2016).

For tobacco dependence, ASSIST_T_ demonstrated weaker but still meaningful discriminatory performance. The most accurate cutoffs for identifying dependence were 5.5 and 6.5, with the lower value offering slightly better sensitivity according to the Youden index. These cutoffs also fall well below the manual’s high-risk threshold of 27. Rather than contradicting the manual guidance, our findings suggest that clinically relevant dependence may manifest at lower levels of tobacco use in mental health populations, especially when early detection is prioritized. The subscale also effectively distinguished moderate from low dependence, with optimal thresholds at 8.5 and 10.5; again, the lower cutoff favors sensitivity. These findings support the utility of the ASSIST in capturing severity gradients in tobacco use, consistent with its dimensional, risk-oriented framework.

In clinical settings that prioritize early intervention, our findings align with contemporary public health perspectives, such as those of Batra et al. (2021) or Anderson et al. (2023), which emphasize that no level of tobacco or alcohol use can be considered risk-free. As individuals with mental disorders may be more susceptible to developing alcohol or tobacco use disorders even at lower levels of consumption (Anker et al., 2023, Swendsen et al., 2010), there is a clear need for sensitive screening tools that can detect early-stage or subthreshold use. This supports the shift toward a dimensional understanding of substance use in the DSM-5, which, unlike the ICD-10, replaces the separate categories of abuse and dependence with a single Substance Use Disorder diagnosis, defined along a severity continuum (American Psychiatric Association, 2013, Hasin et al., 2013). Dimensional diagnostic systems acknowledge that harmful consequences can emerge at various points along this continuum (Hasin et al., 2013), particularly in populations with heightened vulnerability, such as individuals in outpatient mental health care. From this perspective, the application of lower cutoff scores on screening tools like the ASSIST represents a clinically meaningful adaptation that supports earlier detection and more tailored interventions for both alcohol and tobacco use in these settings. Although the ASSIST may be less suited for identifying binary diagnostic thresholds, it demonstrates strong utility in capturing gradations of substance use severity, thereby aligning well with a dimensional approach to screening and intervention.

### 5.3 Limitations and future directions

Some limitations warrant consideration. First, the validation focused exclusively on the alcohol and tobacco subscales of the German self-report version of the ASSIST, limiting conclusions about the instrument’s psychometric properties for other substances. The present study placed emphasis on these two subscales, as they represent the two most prevalent substances used as well as addictive disorders in outpatient settings. A comprehensive validation of the entire ASSIST, including clinician-administered versions, remains necessary for a complete evaluation. Additionally, while ASSIST_A_ and ASSIST_T_ demonstrated statistically significant associations with ICD-10 diagnoses, the small correlation coefficients suggest that the results regarding the ASSIST alcohol and tobacco subscales’ construct validity should be interpreted with caution, indicating that the ASSIST_A_ and ASSIST_T_ may capture aspects of substance involvement not fully reflected by categorical diagnostic criteria. Moreover, findings may not generalize to younger individuals, inpatient or non-psychotherapeutic populations. A more detailed assessment of sociodemographic data would have been necessary to explore potential moderators of substance use patterns and scale performance.. Therefore, future research should extend the validation of the German ASSIST to additional subscales, broader clinical populations including adolescents and inpatients, and incorporate clinician-administered versions. Our results indicate that lowering cutoff scores may facilitate earlier identification of risky or dependent alcohol and tobacco use, warranting replication in future studies. Longitudinal studies investigating the ASSIST’s sensitivity to changes in substance use over the course of psychotherapy and its predictive validity for treatment outcomes would further enhance its clinical relevance.

### 5.4 Clinical implications and conclusion

The present results have practical significance for psychotherapeutic settings. Outpatient care often lacks the resources or time to implement full diagnostic protocols, despite guideline recommendations (Batra et al., 2021, Frischknecht et al., 2021). The ASSIST alcohol and tobacco subscales offer a feasible and scalable tool to bridge this gap. It allows for the efficient identification of substance-related risk, particularly for alcohol and tobacco use, across a dimensional continuum, which is an important advantage in clinical populations characterized by elevated vulnerability and frequent comorbidities (Swendsen et al., 2010). On a practical level, dimensional identification allows psychotherapists to tailor treatment plans according to individual substance use profiles.

Based on the evidence presented, we recommend the routine implementation of the ASSIST as part of the initial diagnostic process of alcohol and tobacco use in psychotherapeutic care. Its integration could be considered in future practice guidelines. For psychotherapists, ASSIST results can inform early therapeutic decisions: for instance, when patients exceed established cut-off scores, this may indicate the need to address substance use explicitly within the treatment plan, e.g., through motivational enhancement strategies, substance-focused interventions, or, where appropriate, consideration of more intensive or integrated treatment settings (e.g., dual-diagnosis programs or inpatient care). Our findings suggest that lowering cutoff scores for alcohol and tobacco use in outpatient mental health settings may improve screening sensitivity. Standardized screening can thereby support early detection and guide tailored, evidence-informed treatment planning.

## CRediT authorship contribution statement

**Esra Sünkel:** Writing – review & editing, Writing – original draft, Methodology, Formal analysis, Conceptualization. **Alla Machulska:** Writing – review & editing, Writing – original draft, Supervision, Methodology, Conceptualization. **Marie Neubert:** Writing – review & editing, Supervision, Data curation. **Tobias Stalder:** Writing – review & editing, Formal analysis. **Tim Klucken:** Writing – review & editing, Supervision, Project administration, Methodology, Conceptualization.

## Funding

This research did not receive any specific grant from funding agencies in the public, commercial, or not-for-profit sectors.

Consent to participate and publication

Written informed consent to participate was obtained from all participants, who can withdraw at any time without giving a particular reason.

Authors' contributions

ES is the corresponding author of the study. ES, AM, and TK: Conceptualization and Methodology. ES was responsible for the data collection and monitoring. AM, TS, MN, and TK contributed toward the data analysis and result presentation as well as revised the manuscript. All the authors have read and approved the final manuscript.

Declaration of generative AI in scientific writing

During the preparation of this work the authors used *DeepL* in order to improve the readability and language of the manuscript. After using this tool, the authors reviewed and edited the content as needed and take full responsibility for the content of the published article.

## Declaration of competing interest

The authors declare that they have no known competing financial interests or personal relationships that could have appeared to influence the work reported in this paper.

## Data Availability

Data will be made available on request.
